# Editorial: Canine Olfactory Detection

**DOI:** 10.3389/fvets.2020.00100

**Published:** 2020-02-27

**Authors:** Claire Guest, Cynthia M. Otto

**Affiliations:** ^1^Medical Detection Dogs, Milton Keynes, United Kingdom; ^2^Penn Vet Working Dog Center, Philadelphia, PA, United States; ^3^Department of Clinical Sciences & Advanced Medicine, University of Pennsylvania, Philadelphia, PA, United States

**Keywords:** olfaction, detection, behavior, scent, working dogs

Throughout history, dogs have fulfilled a whole range of different functions of which the number and diversity are continually expanding. Whilst traditionally dogs have been trained to hunt, herd, and guard, more recently canine roles have targeted olfactory and disease detection tasks. Although not the only species with excellent potential to harness the olfactory senses, dogs have remarkable olfactory acuity and their anatomy and physiology have evolved to support dogs in “seeing” the world through their noses. This Research Topic features original studies and reviews relevant to our theme of Canine Olfactory Detection and highlights the use of dogs in olfactory detection with several papers describing novel targets or capabilities of dogs. In addition, factors that influence canine olfactory detection, from availability of dogs, to training, behavior, medications/interventions, and disease round out the collection.

The expanding list of odors that dogs have been trained to identify raises new opportunities to improve human health and safety. Francis et al. describe a potential new application in narcotic detection to locate illegal synthetic cathinones or “bath salts.” “Bath salts” have variable compositions, induce psycho-stimulant effects and are increasingly being abused (1). This is the first published study to explore whether “bath salts” can be detected by dogs. Certified narcotics detection dogs, which had never been exposed to the odor of “bath salts” were unable to reliably alert to the presence of cathinones, suggesting these compounds do not share volatiles with narcotics on which dogs are commonly trained. However, dogs “imprinted” on the odor reliably alerted to the presence of cathinone and generalized from the trained compound to other “bath salts.” Head space analysis of different “bath salts” suggests some common volatile compounds (e.g., methylone) which may be candidates for the development of a synthetic training aid for “bath salts.”

Medical detection is a growing field for canine olfaction and holds promise for new insights into disease etiology and diagnosis. Fischer-Tenhagen et al. in a proof of concept study, trained two dogs to distinguish breath samples from patients with lung cancer from those from healthy controls and then tested the dogs using synthetic air samples fortified with 1-butanol, 2-butanone, 2-pentanone, and hexanal (compounds increased in lung cancer sufferers) (2). The dogs alerted to three of the four synthetic samples tested. The low sample size limits the conclusions, but this study does suggest that dogs have the potential to verify biomarkers. This is an area of growing interest particularly in the field of medical detection where, long-term, diagnostics using dogs may be inappropriate or unlikely because of the number of tests required due to disease prevalence or complex etiology in the human population. Validation of biomarkers could assist in the rapid development of bio-electronic (E)-noses in the future. Trained dogs could assist in the identification of valid molecules or biomarkers associated with complex disease.

One of the greatest challenges in medical detection is the low concentration of disease associated volatiles, in a background of “normal” volatile compounds present in liquid samples (e.g., blood, urine, sputum). Efforts to determine the limits of canine olfactory detection may help identify dogs with superior potential for medical detection and may help monitor the day to day reliability of the trained dogs. Concha et al. present the first published study to measure limits of detection of known odorants in the fluid phase. Although subject to individual variability, dogs can detect concentrations as low as 1.5 parts per trillion. This detection sensitivity is pertinent if dogs are to assist in the validation of molecules or markers of disease; the markers can be added to liquid background to simulate urine or other fluids for presentation and validation. Selected substances could even be used to “spike” healthy control samples. Dogs trained to assist in this validation task would need to be able to generalize confidently whilst maintaining a high sensitivity, as it is unlikely that the first molecules tested would represent the complete disease signature.

Using functional magnetic resonance imaging (fMRI) on awake unrestrained dogs, Ramaihgari et al. were able to show that zinc nanoparticles can enhance olfactory sensitivity, potentially upregulating both activity and connectivity (3). This provides an explanation for previously reported enhancement in the odor detection capability in the presence of zinc nanoparticles. Behavioral studies are now needed to confirm the findings but potentially these zinc nanoparticles could be used to improve detection capabilities, particularly in environments where very low concentrations of odorants might not otherwise be detected. This possible amplification of the olfactory signal is of great significance to future work and could assist in numerous complex detection tasks. However, further research is necessary to determine if amplification is beneficial or practical.

As applications of canine olfactory ability expand, it is imperative that the value of these dogs is objectively assessed, and their potential capabilities are optimized. We need to optimize accuracy, performance, and welfare in these working dogs. Olfactory performance relies on both the olfactory anatomy (including olfactory receptors, neurons, and olfactory bulb) and the behavior of the dog to communicate the information. Dogs are quick to learn patterns of behavior and past patterns will influence future performance. Typically, we use this to our advantage in training a dog to recognize and respond to an odor of interest; however sometimes the dogs try to “game the system.” Some dogs find the process of searching to be the most valuable reward and if a particular cue leads them to anticipate that they will no longer be able to continue searching, their performance may suffer. Topoleski et al. discuss how these cues which signal the end of a training or testing session are often overlooked by handlers or trainers and may in fact negatively affect the performance of the detector dog. After a complete evaluation of the dog's performance and health, strategies to overcome learned associations may be a valuable tool to improve performance.

With increasing opportunities for employment of detection dogs, there is growing demand for dogs that possess the characteristics for the required tasks. In the United States, many agencies are experiencing a shortage of dogs with the necessary traits for successful olfactory detection (Leighton et al.). In order to identify dogs with the potential for different types of detection work, the working phenotype needs to be clearly and quantitatively defined. As such the selection of dogs with the optimal physical, genetic, and behavioral characteristics is imperative.

Whilst the knowledge of genetics increases, it is essential to address the importance of rearing and training environments. Two papers in this series explore behavioral differences in different types of detection dogs [i.e., search-and-rescue (SAR) and explosive detection dogs]. Behavioral traits such as trainability, fearlessness, and energy are often cited as required for dogs to succeed in search-and-rescue. Hare et al. used the Canine Behavioral Assessment and Research Questionnaire (CBARQ) tool to compare the reported behavior of SAR dogs with a breed-matched sample of pet dogs. SAR dog handlers rated their dogs to have significantly higher trainability and energy, and lower aggression and fear than pet dogs. This study, however, was unable to determine whether the reported differences were the result of underlying personality differences on the basis of which the SAR dogs were initially selected or were a result of rearing and training. It is also not known if the behavioral traits translate to measurable differences in performance. Prospective behavioral and performance studies of dogs specifically bred for detection work are essential to select for dogs with the greatest potential to address the working dog shortage (Leighton et al.).

Lazarowski et al. describe the behavioral evaluation of 146 dogs in an explosive detection dog breeding program. The authors define the phenotype of success in dogs trained to detect and alert to target odors in the aerodynamic wakes of moving persons (“Vapor Wake”). Subjective evaluation scores differed between the 63% of dogs which were successfully trained for detection of person born explosives (e.g., “Vapor Wake,” hand carried, and body worn explosives), and the 17% that were deemed more suited to traditional explosives work or the 20% rejected for any type of detection work. Performance-related measures such as “hunt” and “focus” distinguished the “Vapor Wake” dogs. Environmental traits, often described as fearfulness, distinguished failures from successes but not between “Vapor Wake” and traditional explosives dogs. As shown in previous studies (4), fearfulness can be predicted early in life and has a strong genetic component, it is therefore imperative to select and breed against.

Future studies will be strengthened by more objective and validated measures of these traits. Development of common language and clear phenotype is necessary to increase the success and availability of detection dogs. A multi-disciplinary team of authors representing a variety of expert stakeholders suggest a solution to the current shortage of detection dogs in the USA (Leighton et al.). A “Detector Dogs Center of Excellence” would serve as a national resource for governmental, military and law enforcement working dog agencies to utilize as a data collection and genetic evaluation center. The research goals would be to define quantitative traits involved in odor detection, to better understand how these traits develop, and to evaluate methods to optimize breeding, raising and training detection dogs across all disciplines. This model demonstrates the potential value of a truly collaborative approach.

With an alternative view, perhaps addressing the problem of a shortage of detection dogs, Prada and Furton review the biology and natural olfactory capabilities of birds, suggesting that birds represent a plausible avenue of olfactory detection and urging the research community to consider their use. The use of birds in forensic detection would take advantage of natural avian behaviors including use of odor gradients for navigation and food location. In addition, chickens have provided insight into chemosensory learning and early odor exposure. Although birds may not become common screening agents in airports, they should not be overlooked as a species that can contribute to our understanding of odor detection and may be employed in specific areas.

Despite dramatic progress in understanding areas where canine olfaction can be leveraged for enormous value, there are still many known and unknown factors that could impact canine detection performance. Jenkins et al. provide a much-needed review of the existing literature on the potential effects of health, management (including diet), and microbiota on olfactory performance of dogs. Although few studies have evaluated the effects in dogs, the authors highlight evidence from other species to identify factors that could impact canine olfactory performance. The influence of the microbiome is a growing and fascinating field which is becoming increasingly studied in many areas of biology, performance, and health. This is a fast-growing area which could have major significance for both the canine detector and, in relation to biological or medical detection, may affect the odor signature itself. It is now thought likely that the microbiome is altered by changes in health and wellness of an organism. The authors also highlight the potential beneficial effects of fasting. However, although investigating the timing of feeding and its effect on working ability is an important area of future research it will be vital that dog welfare is prioritized. Metronidazole is a common drug for the treatment of diarrhea; at high doses in some dogs, olfactory abilities are impaired (5). Understanding the potential chemical, environmental, and physiologic factors that could either enhance or impair performance is a critical next step to developing our knowledge of canine olfaction.

The studies in this Research Topic have touched on areas that are essential to further our understanding of the role and performance of working detection dogs. As commented earlier, as canine olfactory usage expands, it is imperative that the value of these dogs is objectively assessed and that their potential capabilities are optimized. The role of the dog as a detector may in the future have many applications, and research such as that published in this Research Topic will assist in ensuring that we use these abilities to further optimize accuracy, performance, and welfare in these working dogs.

## Author Contributions

CG and CO contributed to the content and style of the editorial.

### Conflict of Interest

The authors declare that the research was conducted in the absence of any commercial or financial relationships that could be construed as a potential conflict of interest.
